# Comparative efficacy and safety of sodium–glucose cotransporter 2 inhibitors for renal outcomes in patients with type 2 diabetes mellitus: a systematic review and network meta-analysis

**DOI:** 10.1080/0886022X.2023.2222847

**Published:** 2023-09-19

**Authors:** Junhua Ma, Jiancan Lu, Peiling Shen, Xuemei Zhao, Hongling Zhu

**Affiliations:** Department of Endocrinology, Gongli Hospital of Shanghai Pudong New Area, Shanghai, China

**Keywords:** Sodium-glucose cotransporter-2 inhibitors, type 2 diabetes mellitus, network meta-analysis, meta analysis

## Abstract

In this study, the summarized WMDs and RRs were calculated using a pairwise analysis and a network meta-analysis with a random effects model, to compare and rank the efficacy and safety of SGLT-2i for renal outcomes in patients with T2DM. Among 1894 identified articles, 30 trials including 50,244 patients with T2DM were evaluated. Network analysis revealed that the administration of canagliflozin was associated with a reduced risk of renal impairment (surface under the cumulative ranking: 90.8%). Further, although the administration of SGLT-2i was not associated with the risk of renal impairment (RR = 0.88, 95%CI = 0.68–1.15, *p* = 0.354), the administration of empagliflozin was associated with a reduced risk of renal impairment compared to that with the administration of placebo (RR = 0.74, 95%CI = 0.62–0.90, *p* = 0.002). Moreover, compared with the administration of a placebo, the administration of 50, 100, and 200 mg of canagliflozin was associated with lower serum creatinine levels. Furthermore, compared with the administration of a placebo, the administration of 100 mg canagliflozin, 2.5 mg dapagliflozin, and 25 mg empagliflozin was associated with a lower reduction in the estimated glomerular filtration rate. Except for 300 mg canagliflozin, all SGLT-2i were associated with greater increases in blood urea nitrogen levels (WMD = 1.39, 95%CI = 1.20–1.59, *p* < 0.001). Finally, the administration of all SGLT-2i significantly increased the ratio of urinary glucose to creatinine compared with the ratio upon administration of placebo (WMD = 36.21, 95%CI = 31.50–40.92, *p* < 0.001). Briefly, canagliflozin exerts the greatest therapeutic effect in terms of reducing the risk of renal impairment. Empagliflozin and canagliflozin may be more effective than other SGLT-2i in preventing renal impairment.

## Introduction

Type 2 diabetes mellitus (T2DM) is a chronic disease caused by complex genetic and environmental factors in which blood glucose control is crucial for patient treatment and management [1]. As the fifth leading cause of disability in 2017 [2], it places considerable socioeconomic pressure on patients and a significant burden on global health economies, with estimated costs of US$825 billion [3]. Notably, obesity, hypertension, dyslipidemia, and cardiovascular disease can cause considerable morbidity and mortality in patients with T2DM [4–6]. Complications of T2DM have traditionally been classified into microvascular and macrovascular diseases, including retinopathy, nephropathy, neuropathy, coronary heart disease, cerebrovascular disease, and peripheral vascular diseases, which are responsible for significant morbidity [7]. According to the treatment guidelines for T2DM, glucose control remains a key focus in the management of patients with T2DM. However, according to data from the United States National Health and Nutrition Examination Survey and the Behavioral Risk Factor Surveillance System, in the past decade, blood glucose, blood pressure, and serum lipid controls could not be achieved in 33.4%–48.7% of patients with T2DM [8]. Furthermore, owing to the heterogeneous molecular etiology of T2DM, available hypoglycemic drugs that exert effects through different mechanisms often fail to adequately control blood glucose even when used in combination [9,10].

According to a previous study, the use of available drugs for diabetes enabled 53% of patients to achieve glycated hemoglobin (HbA1c) levels of <7.0% [11]. Notably,sodium–glucose cotransporter 2 inhibitors (SGLT-2i) are novel oral drugs for T2DM and are effective in lowering both HbA1c and blood glucose levels. Moreover, they are known to have a favorable safety profile and are associated with a low risk of hypoglycemia, regardless of their use as monotherapy or combination therapy [12]. Following the controversy associated with the cardiovascular safety of rosiglitazone in 2007, antidiabetic drugs have been developed with therapeutic models prioritizing cardiovascular safety [13]. SGLT2i are commonly used to reduce the risk of arteriosclerotic cardiovascular disease (ASCVD); decrease urinary protein levels; preserve renal function; and improve metabolic indices, such as obesity, hypertension, hyperuricemia, and hyperlipidemia. In addition, the insulin-independent features of SGLT2i support their administration throughout the natural progression of T2DM [12]. Notably, SGLT2i are ranked as first-line drugs over metformin for patients with T2DM complicated by ASCVD, heart failure, or chronic kidney disease, regardless of their baseline HbA1c levels and individual targets. A consensus statement from the American Diabetes Association, European Association for the Study of Diabetes, and Chinese Diabetes Society recommends the administration of glucagon-like peptide-1 receptor agonists or SGLT-2i in patients whose blood glucose levels are inadequately controlled with metformin [14].

Although several systematic reviews have examined the role of SGLT-2i in patients with T2DM [15–21], to the best of our knowledge, no study has provided quantitative data regarding the efficacy and safety of SGLT-2i for renal outcomes in patients with T2DM or a comparison and ranking of the types of SGLT-2i based on direct or indirect evidence. SGLT-2i significantly reduces glucose reabsorption with the increased sodium concentration in the macula densa, which was associated with lower renal energy demand and oxygen consumption. Moreover, SGLT-2i could reducing oxidative stress and increase renal oxygen delivery [22]. Previous large multicenter clinical trials aimed to intensively lower blood glucose levels, and their results indicated that intensive glucose control measures could improve the prognosis of microvascular complications (including two-times higher serum creatinine levels, end-stage renal disease [ESRD], and death due to renal diseases) [23–25]. Further, owing to the lack of evidence from direct comparisons, network meta-analysis (NMA) is the methodology of choice for synthesizing data, which helps select the treatment of interest using indirect comparisons. Therefore, this systematic review and NMA aimed to compare different types of SGLT-2i at various doses based on renal outcomes in patients with T2DM.

## Materials and methods

### Patient and public involvement

This meta-analysis included no patient or public involvement; hence, ethics approval and consent for participation were not required.

### Search strategy and selection criteria

This systematic review and NMA was conducted in accordance with the Preferred Reporting Items for Systematic Reviews and Meta-Analysis [26]. Placebo-controlled or head-to-head randomized controlled trials (RCTs) of six SGLT-2i were included in the study. The electronic databases MEDLINE, EMBASE, and Cochrane Library were searched from their inception to 20 March 2023, without any restrictions in terms of publication language or status. The key search terms were ‘diabetes mellitus’ OR ‘type 2 diabetes’ OR ‘type ii diabetes’ combined with the names of all SGLT-2i (See Supplemental online material 1 [section 1]). Notably, clinicaltrials.gov was searched to identify completed studies with unpublished data. Reference lists of relevant original articles and reviews were manually checked to identify studies that met the inclusion criteria. When data were missing for studies that met the inclusion criteria, we contacted the corresponding author of the original article for additional information.

Two reviewers (MJ and LJ) performed the literature search and selected studies based on a standardized approach. Further, a third reviewer (ZH) resolved disagreements until a mutual consensus was reached among the three reviewers. Studies were included if they met the following patient, intervention, control, outcome, and study-design criteria: 1) studies in which all patients were diagnosed with T2DM; 2) studies involving administration of SGLT-2i, including canagliflozin, dapagliflozin, empagliflozin, ertugliflozin, ipragliflozin, and tofogliflozin; 3) studies involving comparison with placebo or SGLT-2i; 4) studies in which the primary endpoint was mild renal impairment and the secondary endpoints were serum creatinine level, estimated glomerular filtration rate (GFR), serum blood urea nitrogen (BUN) level, and urinary glucose/creatinine ratio; and 5) RCTs.

### Data collection and quality assessments

Two reviewers (MJ and SP) applied a standardized flow to extract all relevant information from the included studies. Data regarding the following items were collected: first author’s name, publication year, clinical trial registration number, country, sample size, mean age of participants, the proportion of male participants, baseline body mass index, T2DM duration, baseline HbA1c levels, intervention, control, renal impairment definition, and investigated outcomes. Notably, the quality of each trial was evaluated using version 2 of the Cochrane risk of bias tool in which the bias domains include biases caused by the randomization process (low risk/some concerns/high risk), deviations from the intended interventions (low risk/some concerns/high risk), and missing outcome data (low risk/some concerns/high risk) as well as biases in the measurement of the outcomes (low risk/some concerns/high risk), selection of the reported results (low risk/some concerns/high risk), and overall bias (low risk/some concerns/high risk). Furthermore, two reviewers (SP and ZX) independently assessed the quality of each trial, and a third reviewer resolved any conflicts between the two reviewers. Disagreements between the reviewers were resolved by discussion to reach a consensus.

### Statistical analysis

Among the studies that published data more than once, those that reported the most informative and complete data were selected. The summarized weighted mean differences (WMDs) and relative risks (RRs) with 95% confidence intervals (CIs) were calculated for continuous and dichotomous data, respectively. Pairwise analysis and NMA were performed using the random effects model [27,28]. *I^2^* and Q statistics were used to assess heterogeneity for pairwise meta-analyses. An *I^2^* value of >50% or a *p*-value of <0.10 indicated significant heterogeneity among the included trials [29]. Moreover, to assess the heterogeneity for NMA, we compared the posterior distribution of the estimated heterogeneity variance with its predictive distribution [30]. Further, NMA was used to compare different SGLT-2i *via* indirect and mixed comparisons [31]. A loop-specific approach was then used to assess the differences between direct and indirect estimates for a specific comparison in the loop, which can assess inconsistency [32].

The design-by-treatment interaction inconsistency model was applied to evaluate the assumption of consistency in the entire network [31]. Owing to the heterogeneity among the included patients, we used this inconsistency model to analyze data. The surface under the cumulative ranking (SUCRA) probabilities were used to rank the treatment strategies for each investigated outcome [33]. Publication bias was assessed using comparison-adjusted funnel plots to determine the presence of small-study effects in our analysis [34]. The primary endpoint of the present study was assessed using pairwise comparisons and NMA, and the secondary endpoints were evaluated using pairwise meta-analysis. Moreover, for dichotomous data, treatment strategy comparisons were made based on the type of SGLT-2i. All analyses were performed using STATA software (version 10.0; Stata Corporation, College Station, TX, USA).

## Results

### Literature search

**Figure 1.**
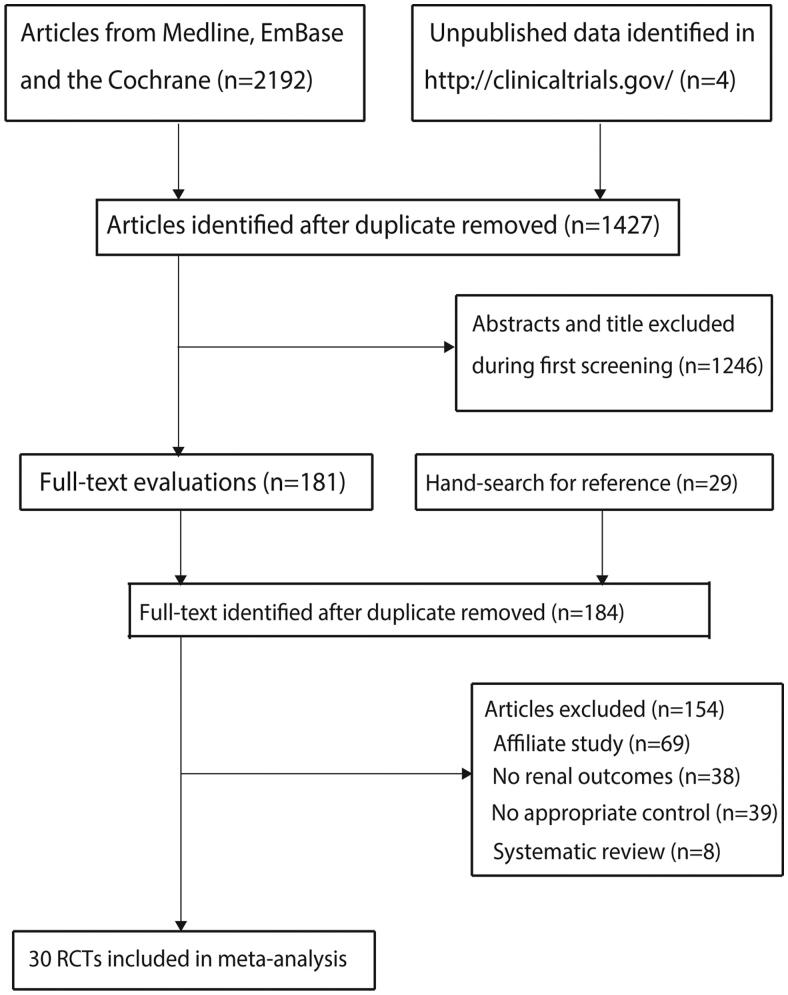
Details of the literature search and study selection process.

Overall, 1894 articles were identified from the literature search; of these, 541 duplicate articles were excluded. Further, 1207 articles with irrelevant titles or abstracts were excluded. The remaining 146 articles were retrieved for full-text evaluation. A manual review of the reference lists revealed 26 articles, of which 24 duplicates were excluded. Thus, 148 articles were retrieved for full-text evaluation; of them, 118 were excluded as follows: affiliate studies—multiple studies with participants belonging to the same RCT (*n* = 43); studies without measurements of renal outcomes (*n* = 34); studies without appropriate control groups (i.e., groups in which no placebo or SGLT-2i are administered) (*n* = 33); and systematic reviews (*n* = 8). Details regarding the literature search and study selection process are presented in Figure 1.

### Study characteristics

Characteristics of the identified studies and patients are presented in Table 1. Overall, 30 RCTs including 50,244 patients with T2DM were identified from 2009 to 2019 [35–64]. Comparisons among six types of SGLT-2i or between SGLT-2i and placebo were included in the final systematic review and NMA. In total, 40–17,160 patients with a mean age of 51.4–65.8 years were included in each trial, and 44.2%–77.1% of patients were men. The baseline HbA1c levels were found to be 7.40%–9.30%. A total of 25 multinational [35–44,46,47,49–54,56–58,60,62–64] and 5 single-nation [45,48,55,59,61] trials were identified. Of them, 7 [42–44,55,58,59,64], 19 [35–41,45,47–50,52–54,57,58,60,62,63], 2 [46,51], and 2 [56,61] trials used canagliflozin, dapagliflozin, empagliflozin, and ipragliflozin as the intervention. The follow-up period ranged from 6 weeks to 4.2 years. Supplemental online material 1 (section 2) presents the quality of the included studies. All 30 RCTs had a low risk of bias associated with the randomization process, missing outcome data, and outcome measurements [35–64]. Nine studies had some concerns regarding the risk of bias associated with the selection of results [38,42–44,47,50,52,53,61]. Based on the assessment for biases associated with deviations from the intended intervention, four studies had some concerns, whereas one study had a high risk [59]. The results of the quality of the included studies are summarized in Supplemental online material 1 (section 2).

**Table 1.** The baseline characteristics of included studies and involved patients.

Study	Register No.	Country	Sample size	Age (years)	Male (%)	BMI (kg/m^2^)	T2DM duration	HbA1c (%)	Intervention	Control	Follow-up duration	Definition of renal impairment
List 2009 [34]	NCT00263276	Multicountries	343	54.2	49.3	31.7	NM	7.80	Dapagliflozin (2.5,5.0,10,20,50 mg)	Placebo	12 weeks	MedDRA 10
Ferrannini 2010 [35]	NCT00528372	Multicountries	558	52.0	49.5	32.6	0.49 yrs	8.30	Dapagliflozin (2.5,5.0,10 mg)	Placebo	24 weeks	MedDRA 11.1
Bailey 2010 [36]	NCT00528879	Multicountries	546	53.9	53.5	31.5	6.1 yrs	8.00	Dapagliflozin (2.5,5.0,10 mg)	Placebo	24 weeks	MedDRA 11.1
Strojek 2011 [37]	NCT00680745	Multicountries	592	59.8	48.1	NM	7.4 yrs	8.10	Dapagliflozin (2.5,5.0,10 mg)	Placebo	24 weeks	MedDRA 12.1
Wilding 2012 [38]	NCT00673231	Multicountries	800	59.3	47.8	33.1	13.6 yrs	8.53	Dapagliflozin (2.5,5.0,10 mg)	Placebo	48 weeks	MedDRA 12.1
Henry 2012 [39]	NCT00643851 and NCT00859898	Multicountries	814	51.8	46.2	NM	1.8 yrs	9.20	Dapagliflozin (5.0,10 mg)	Placebo	24 weeks	MedDRA 12 and 13
Rosenstock 2012 [40]	NCT00683878	Multicountries	420	53.5	49.5	NM	4.1 yrs	8.37	Dapagliflozin (5.0 and 10 mg)	Placebo	48 weeks	NM
Bode 2013 [41]	NCT01106651	Multicountries	716	63.6	55.5	31.6	11.7 yrs	7.70	Canagliflozin (100, 300 mg)	Placebo	26 weeks	NM
Stenlof 2013 [42]	NCT01081834	Multicountries	584	55.4	44.2	31.6	4.3 yrs	8.00	Canagliflozin (100, 300 mg)	Placebo	26 weeks	NM
Wilding 2013 [43]	NCT01106625	Multicountries	469	56.8	51.0	33.1	9.6 yrs	8.10	Canagliflozin (100, 300 mg)	Placebo	26 weeks	NM
Kaku 2013 [44]	NCT00972244	Japan	279	57.3	77.1	NM	4.8 yrs	8.10	Dapagliflozin (1,2.5,5.0,10 mg)	Placebo	12 weeks	MedDRA
Kovacs 2014 [45]	NCT01210001	Multicountries	498	54.5	48.4	29.2	NM	8.10	Empagliflozin (10,25 mg)	Placebo	24 weeks	MedDRA 15.0
Leiter 2014 [46]	NCT01042977	Multicountries	962	63.7	67.0	32.8	13.2 yrs	8.10	Dapagliflozin (10 mg)	Placebo	24 weeks	MedDRA 12.1
Kaku 2014 [47]	NM	Japan	261	58.8	59.4	25.4	4.9 yrs	7.47	Dapagliflozin (5,10 mg)	Placebo	24 weeks	NM
Ji 2014 [48]	NCT01095653	Multicountries	393	51.4	65.4	25.6	1.4 yrs	8.26	Dapagliflozin (5,10 mg)	Placebo	24 weeks	MedDRA 15.0
Matthaei 2015 5[49]	NCT01392677	Multicountries	216	61.0	49.1	32.0	9.5 yrs	8.16	Dapagliflozin (10 mg)	Placebo	24 weeks	MedDRA 15.1
Wanner 2016 [50]	NCT01131676	Multicountries	7018	63.1	71.4	30.7	NM	8.07	Empagliflozin (10,25 mg)	Placebo	3.1 yrs	MedDRA
Yang 2016 [51]	NCT01095666	Multicountries	444	53.7	54.3	26.1	4.9 yrs	8.13	Dapagliflozin (5,10 mg)	Placebo	24 weeks	MedDRA 16.0
Weber 2016 [52]	NCT01137474	Multicountries	613	55.9	57.1	NM	7.9 yrs	8.00	Dapagliflozin (10 mg)	Placebo	12 weeks	NM
Weber 2016 [53]	NCT01195662	Multicountries	449	56.5	55.0	NM	7.5 yrs	8.10	Dapagliflozin (10 mg)	Placebo	12 weeks	International Conference on Harmonization Good Clinical Practice criteria
Townsend 2016 [54]	NM	US	169	58.6	58.0	33.3	NM	8.10	Canagliflozin (100, 300 mg)	Placebo	6 weeks	NM
Lu 2016 [55]	NCT01505426	Multicountries	170	53.7	45.3	26.8	6.2 yrs	7.74	Ipragliflozin (50 mg)	Placebo	24 weeks	MedDRA 14.0
Frías 2016 [56]	NCT02229396	Multicountries	455	54.0	47.9	32.6	7.5 yrs	9.30	Dapagliflozin (10 mg)	Placebo	28 weeks	MedDRA 18.1
Neal 2017 [57]	NCT01032629 and NCT01989754	Multicountries	10142	63.3	64.2	32.0	13.5 yrs	8.20	Canagliflozin (100 to 300 mg)	Placebo	3.6 yrs	Requirement for renal replacement therapy and doubling of serum creatinine, MedDRA
Takashima 2018 [58]	UMIN000031454	Japan	40	65.1	57.5	25.2	NM	7.40	Canagliflozin (100 mg)	Placebo	52 weeks	NM
Fioretto 2018 [59]	NCT02413398	Multicountries	321	65.8	56.7	32.1	14.4 yrs	8.18	Dapagliflozin (10 mg)	Placebo	24 weeks	MedDRA
Han 2018 [60]	NCT02452632	Korea	139	57.5	49.6	25.8	11.4 yrs	7.91	Ipragliflozin (50 mg)	Placebo	24 weeks	NM
Yang 2018 [61]	NCT02096705	Multicountries	272	57.5	47.8	26.5	12.5 yrs	8.55	Dapagliflozin (10 mg)	Placebo	24 weeks	MedDRA 18.1
Wiviott 2019 [62]	NCT01730534	Multicountries	17160	63.9	62.6	32.1	10.5 yrs	8.30	Dapagliflozin (10 mg)	Placebo	4.2 yrs	NM
Mahaffey 2019 [63]	NCT02065791	Multicountries	4401	63.0	66.1	31.3	15.8 yrs	8.30	Canagliflozin (100 mg)	Placebo	2.6 yrs	NM

### NMA

**Figure 2.**
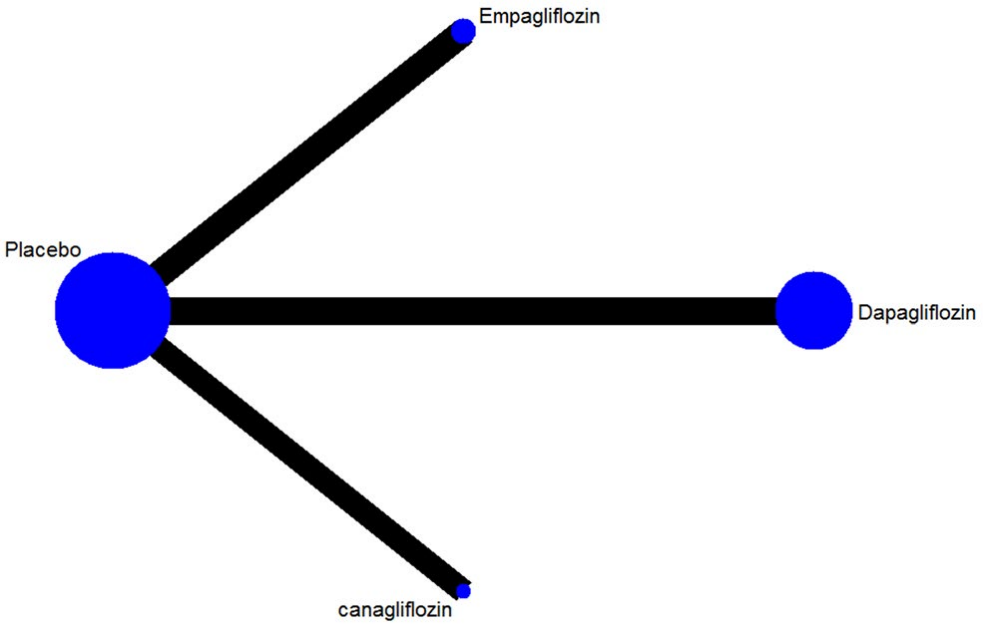
Network of comparisons of renal impairment included in the analysis.

**Figure 3.**
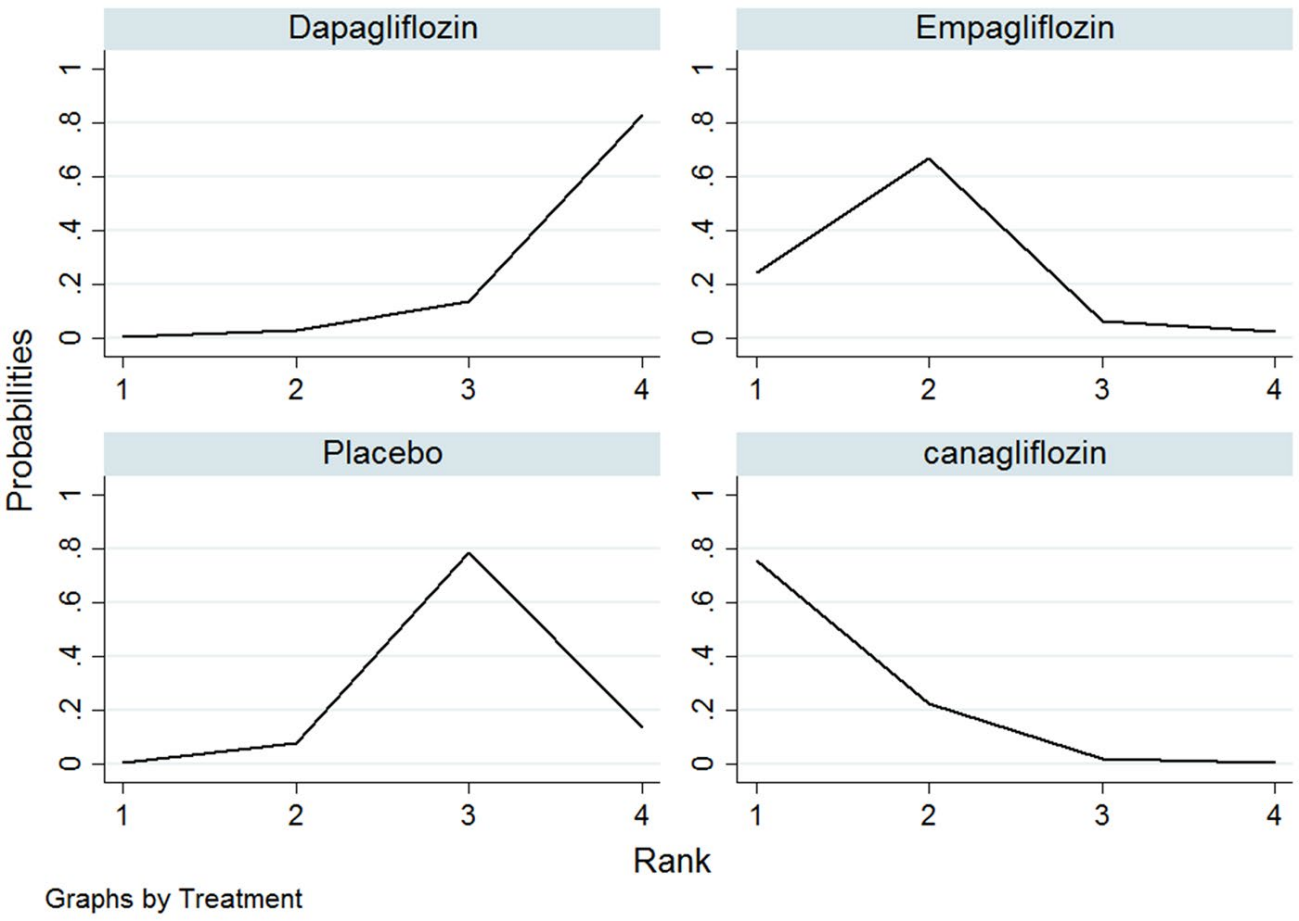
Surface under the cumulative ranking curve (SUCRA) test for renal impairment. The X-axis indicated the ranking of treatment, whereas the Y-axis indicated the probability ranking or better.

**Figure 4.**
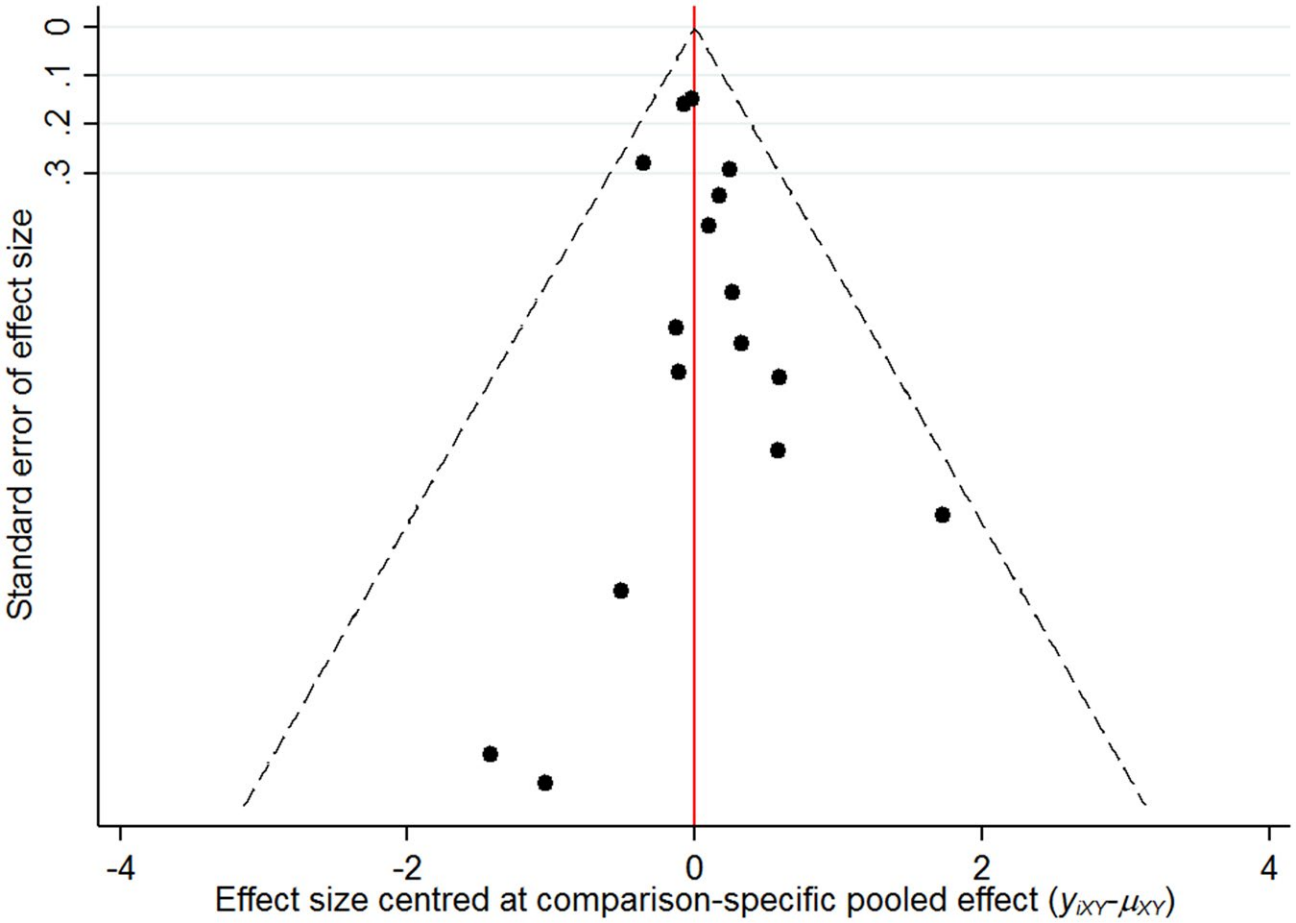
Funnel plot for renal impairment.

The networks of eligible comparisons for the risk of renal impairment in NMA are summarized in Figure 2. The SUCRA probabilities indicated that canagliflozin (SUCRA: 90.8%) and empagliflozin (SUCRA: 71.0%) were associated with the lowest risk of renal impairment (Figure 3). Network analysis revealed that canagliflozin was associated with a lower risk of renal impairment than dapagliflozin (RR: 0.47; 95% CI: 0.24–0.91) or placebo (RR: 0.56; 95% CI: 0.32–0.98); conversely, the results of other comparisons were not statistically significant (Table 2). Funnel plot was relatively symmetrical, and there was no evidence of publication bias for renal impairment (Figure 4) (Supplemental online material 1 [section 3]).

**Table 2.** Details of the pairwise comparisons for agents in terms of renal impairment.

	Dapagliflozin	Empagliflozin	Placebo	Canagliflozin
Dapagliflozin	–	0.60 (0.34–1.06)	0.83 (0.58–1.19)	0.47 (0.24–0.91)
Empagliflozin	1.67 (0.95–2.94)	–	1.38 (0.90–2.13)	0.78 (0.38–1.57)
Placebo	1.21 (0.84–1.74)	0.72 (0.47–1.11)	–	0.56 (0.32–0.98)
Canagliflozin	2.15 (1.10–4.17)	1.29 (0.64–2.60)	1.78 (1.02–3.10)	–

### Direct meta-analysis

The administration of empagliflozin was associated with a lower risk of renal impairment than the administration of placebo (RR: 0.74; 95% CI: 0.62–0.90; *p* = 0.002) (See Supplemental online material 1 [section 3]). The administration of 50 mg (WMD: −1.86; 95% CI: −2.24 to −1.48), 100 mg (WMD: −2.30; 95% CI: −2.70 to −1.90), and 200 mg (WMD: −1.59; 95% CI: −1.94 to −1.24) canagliflozin was associated with a greater reduction in serum creatinine levels than the administration of placebo. However, the administration of 2.5 mg dapagliflozin was associated with higher serum creatinine levels than the administration of placebo (WMD: 0.75; 95% CI: 0.02–1.47) (See Supplemental online material 1 [section 4]). The results of the pairwise meta-analysis for the change in estimated GFR between SGLT-2i and placebo are shown in Supplemental online material 1 (section 5). Notably, the administration of 2.5 mg dapagliflozin (WMD: −1.50; 95% CI: −1.71 to −1.29) and 25 mg empagliflozin (WMD: −1.62; 95% CI: −2.68 to −0.57) was associated with a greater reduction in estimated GFR, whereas the administration of 100 mg canagliflozin was associated with significantly increased estimated GFR compared with the administration of placebo (WMD: 4.10; 95% CI: 3.33–4.87). The results of the pairwise meta-analysis for the change in BUN levels between SGLT-2i and placebo are shown in Supplemental online material 1 (section 6). Most SGLT-2i were associated with higher BUN levels than placebo, with WMDs ranging from 0.68 (95% CI: 0.54–0.82) for 200 mg canagliflozin to 2.28 (95% CI: 2.12–2.44) for 50 mg dapagliflozin; however, the BUN level associated with 300 mg canagliflozin was not statistically significant compared to that associated with placebo. Furthermore, all dapagliflozin doses were associated with a higher urinary glucose/creatinine ratio. The WMD values ranged from 28.20 (95% CI: 16.16–40.24) for 2.5 mg dapagliflozin to 42.71 (95% CI: 37.28–48.13) for 10 mg dapagliflozin (See Supplemental online material 1 [section 7]).

## Discussion

This comprehensive quantitative systematic review and NMA was based on 30 RCTs including 50,244 patients with T2DM randomly assigned to receive treatment with six different SGLT-2i or placebo. The findings of this study expand the findings of previous systematic reviews that investigated the role of SGLT-2i in cardiovascular or specific adverse events [15–21]. Notably, the present study is more comprehensive because six different SGLT-2i and a placebo were evaluated in the study, and the results revealed that canagliflozin exerts the greatest therapeutic effect in terms of reducing the risk of renal impairment. Moreover, empagliflozin was found to be associated with a reduced risk of renal impairment. Compared with placebo, canagliflozin doses of 50, 100, and 200 mg were associated with lower serum creatinine levels. Moreover, compared with placebo, 100 mg canagliflozin, 2.5 mg dapagliflozin, and 25 mg empagliflozin were associated with reduced estimated GFR. Furthermore, except for canagliflozin 300 mg, all SGLT-2is were associated with higher BUN levels. The administration of all SGLT-2i significantly increased the urinary glucose/creatinine ratio compared with the administration of placebo.

Safety analyses revealed that canagliflozin and empagliflozin have the potential to provide protection against renal impairment. Moreover, we found that canagliflozin was associated with a lower risk of renal impairment than dapagliflozin and placebo. A previous NMA by Lin et al. found that SGLT-2i have favorable renal protective effects; however, their study did not find any significant differences in the effects between different SGLT-2i [65]. This inconsistency in the results could be attributed to the fact that in our study, renal impairment was compared among SGLT-2i based on the type of drugs, whereas in the previous meta-analysis, it was compared based on the type and dose of SGLT-2i. Moreover, in our study, the numbers of trials on each treatment were higher than those in the previous meta-analysis, and the power of our study was stronger to detect small differences among SGLT-2i. Furthermore, it has been reported that the beneficial effects of canagliflozin are potentially independent of glucose levels and may be attributed to a reduction in intraglomerular pressure [66–68]. Empagliflozin could also reduce hyperglycemia in patients with T2DM by reducing the renal reabsorption of glucose to increase urinary glucose excretion [69]. In addition, SGLT-2i could act through natriuresis, blood pressure reduction, improved tubular glomerular feedback, vascular compliance, and endothelial function. Finally, the definition of renal impairment for dapagliflozin varies among the included studies, possibly explaining the nonsignificant differences between dapagliflozin and control in terms of the risk of renal impairment [70,71].

We found that 2.5 mg dapagliflozin and 25 mg empagliflozin were associated with a greater reduction in estimated GFR, whereas 100 mg canagliflozin was associated with significantly increased estimated GFR compared with placebo. These results are inconsistent with those of the DAPA-CKD and EMPA-KIDNEY trials, suggesting renal protective effects of dapagliflozin and empagliflozin [72,73]. The beneficial effects of SGLT-2i may be attributed to the reduction in intraglomerular pressure, which could be mediated by natriuresis and glucose-induced osmotic diuresis [74,75]. The inconsistency in the results between our study and previous trials could be explained by the different roles of SGLT-2i in patients with or without chronic kidney disease. Moreover, compared with placebo, 50, 100, and 200 mg canagliflozin doses were associated with lower serum creatinine levels. This finding could be attributed to a reduction in the intraglomerular pressure independent of glucose levels [66]. Furthermore, we found that compared with placebo, most SGLT-2i were associated with higher BUN levels. However, in only a small number of trials, these results were reported according to the type and dose of SGLT-2i; thus, further trials are warranted. Moreover, osmotic diuresis was found to be associated with increased urinary glucose excretion after the administration of SGLT-2i, which might play an important role in increased BUN levels [76].

This study has some limitations. First, most of the identified studies investigated the effects of SGLT-2i on cardiovascular and metabolic factors, whereas only a few studies reported the incidence of renal impairment. Second, the definition of renal impairment varied among the included studies, which is possibly affected by WMDs from the overall analysis. Third, substantial heterogeneity was noted for continuous data, and further subgroup analyses based on countries or continents, race, or follow-up duration are needed. Fourth, although the results of NMA suggested the best treatment option for patients with T2DM, the efficacy of each treatment strategy was not balanced. Fifth, this study was not registered online, and the transparency of the study was limited. Finally, the inherent limitations for any meta-analysis related to published articles, including multiple comparisons according to patient characteristics, and publication bias should be noted.

The results of this study provide comprehensive evidence for the optimal use of SGLT-2i in patients with T2DM to improve renal outcomes. Moreover, the study found that canagliflozin and empagliflozin may play protective roles against renal impairment in patients with T2DM.

## Supplementary Material

Supplemental Material

## Data Availability

All data relevant to the study are included in the article and uploaded as supplementary information.
