# Supplementary material for: Comparative efficacy and safety of sodium–glucose cotransporter 2 inhibitors for renal outcomes in patients with type 2 diabetes mellitus: a systematic review and network meta-analysis
Source: Ren Fail. 2023 Sep 19;45(2):2222847. doi: 10.1080/0886022X.2023.2222847 (PMC10512796; doi:10.1080/0886022X.2023.2222847)
Supplement: Supplemental Material [file IRNF_A_2222847_SM1456.pdf]

Supplementary appendix

Contents

1. Search strategies

2. Quality assessment of included studies

3. renal impairment

4. serum creatinine

5. Estimated GFR

6. BUN

7. Urinary glucose/creatinine ratio

Information Classification: General

1. Search strategies

MedLine:

(Diabetes mellitus or type 2 diabetes or type ii diabetes) AND (Sodium glucose cotransporter 2 inhibitor OR sodium glucose transporter 2 inhibitor* or sodium glucose transporter ii inhibitor* or SGLT2 inhibitor* OR Canagliflozin or Dapagliflozin or Empagliflozin or Ertugliflozin or Tofogliflozin or Ipragliflozin or Remogliflozin) AND (randomized controlled trial [Publication Type] OR controlled clinical trial [Publication Type] OR randomized [Title/Abstract] OR placebo [Title/Abstract] OR drug therapy [MeSH Subheading] OR randomly [Title/Abstract] OR trial [Title/Abstract] OR groups [Title/Abstract] NOT animals [mh].

EMBASE:

#1. (Diabetes mellitus or type 2 diabetes or type ii diabetes).af

#2. Sodium glucose cotransporter 2 inhibitor.af or (sodium glucose transporter 2 inhibitor* or sodium glucose transporter ii inhibitor* or SGLT2 inhibitor*).af

#3. (Canagliflozin or Dapagliflozin or Empagliflozin or Ertugliflozin or Tofogliflozin or Ipragliflozin or Remogliflozin).af

#4. #2 OR #3

#5. (randomi*ed controlled trial or randomi*ed trial).af

#6. #1 AND #4 AND #5

Cochrane Library Central Register of Controlled Trials:

#1. Diabetes mellitus or type 2 diabetes or type ii diabetes

#2. (sodium glucose transporter 2 inhibitor* or sodium glucose transporter ii inhibitor* or SGLT2 inhibitor*) or (Canagliflozin or Dapagliflozin or Empagliflozin or Ertugliflozin or Tofogliflozin or Ipragliflozin or Remogliflozin)

#3. #1 AND #2

Information Classification: General

2. Quality assessment of included studies

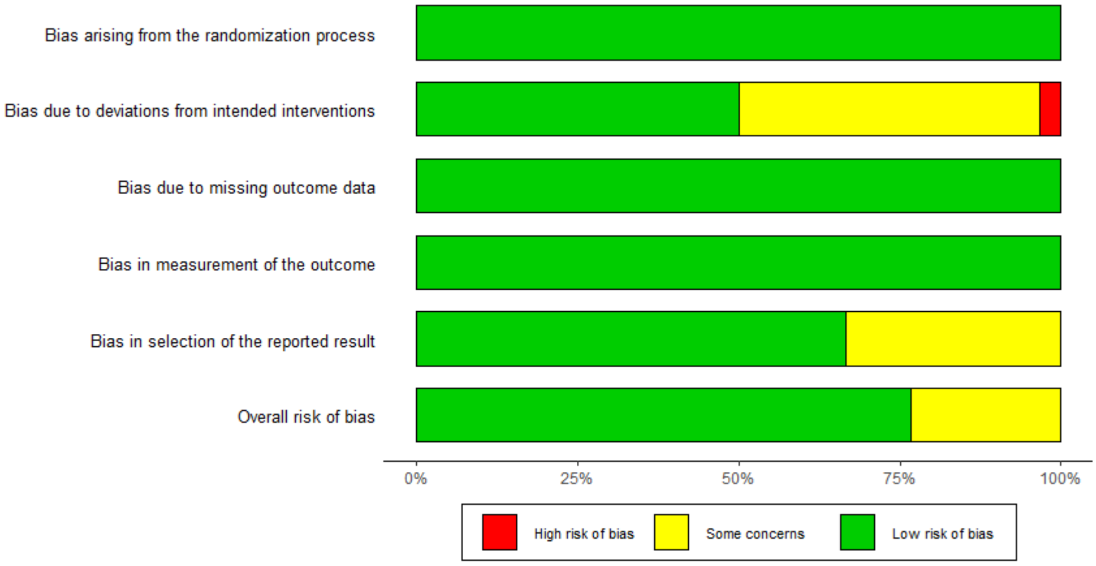

Figure1 Risk of bias graph figure

Information Classification: General

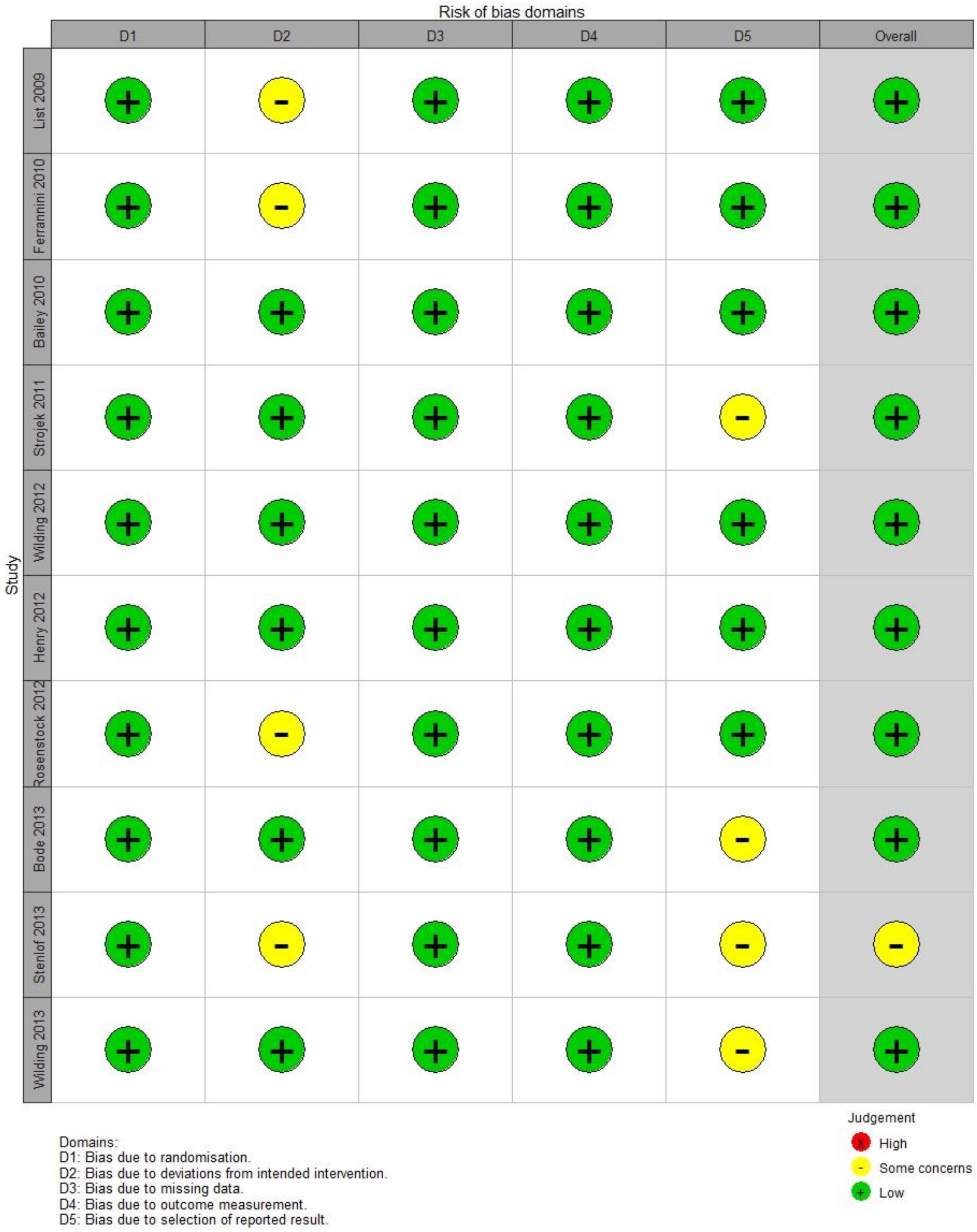

Figure2 Risk of bias of each literature

Information Classification: General

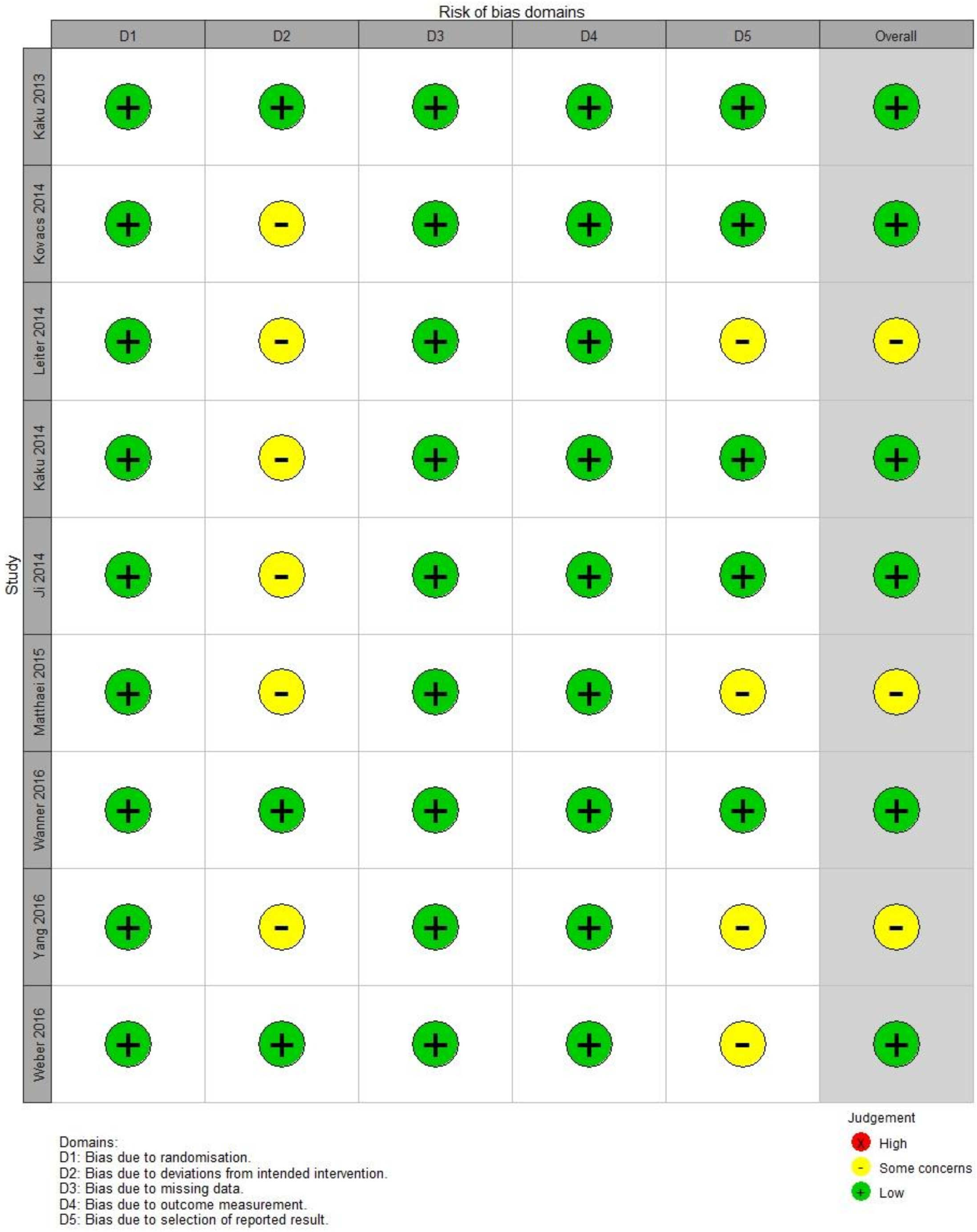

Figure2 (continued) Risk of bias of each literature

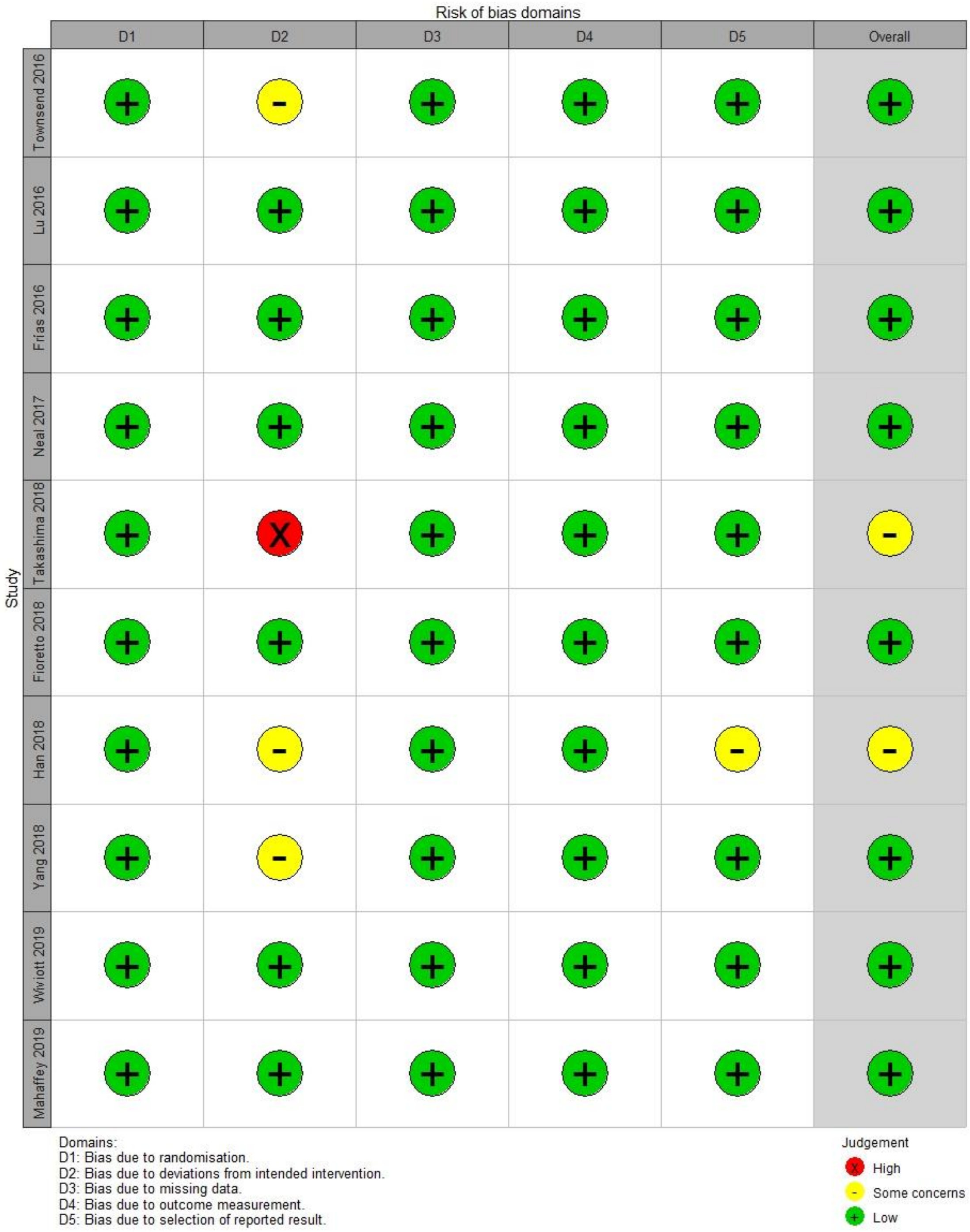

Figure2 (continued) Risk of bias of each literature

3. Renal impairment/failure

Table 1. The summary results for the risk of renal impairment/failure

Drug	No. of cohorts	RR and 95%CI	*P* value	I^2 (%)	*P* value for I^2
Canagliflozin	3	0.58 (0.25 to 1.38)	0.219	90.4	< 0.001
Dapagliflozin	16	1.21 (0.80 to 1.82)	0.375	49.0	0.014
Empagliflozin	5	0.74 (0.62 to 0.90)	0.002	0.0	0.728
Overall	24	0.88 (0.68 to 1.15)	0.354	66.9	< 0.001

4. Serum creatinine (umol/l)

Table 1. The summary results for the changes of serum creatinine (umol/l)

Drugs	Dose	No. of cohorts	WMD and 95%CI	*P* value	I^2 (%)	*P* value for I^2
Canagliflozin	50.0 mg	1	-1.86 (-2.24 to -1.48)	< 0.001	-	-
	100.0 mg	1	-2.30 (-2.70 to -1.90)	< 0.001	-	-
	200.0 mg	1	-1.59 (-1.94 to -1.24)	< 0.001	-	-
	300.0 mg	2	-0.40 (-1.70 to 0.90)	0.543	94.9	< 0.001
Dapagliflozin	2.5 mg	4	0.75 (0.02 to 1.47)	0.044	95.2	< 0.001
	5.0 mg	6	-0.42 (-0.91 to 0.06)	0.087	94.6	< 0.001
	10.0 mg	6	0.32 (-0.28 to 0.92)	0.299	97.1	< 0.001
	20.0 mg	1	-0.88 (-4.00 to 2.23)	0.578	-	-
	50.0 mg	1	1.77 (-1.70 to 5.24)	0.318	-	-
Overall	-	23	-0.25 (-0.69 to 0.18)	0.258	97.9	< 0.001

5. Estimated GFR (ml/min/1.73 m^2)

Table 1. The summary results for the changes of estimated GFR (ml/min/1.73 m^2)

Drugs	Dose	No. of cohorts	WMD and 95%CI	*P* value	I^2 (%)	*P* value for I^2
Canagliflozin	100.0 mg	1	4.10 (3.33 to 4.87)	< 0.001	-	-
Dapagliflozin	2.5 mg	1	-1.50 (-1.71 to -1.29)	< 0.001	-	-
	5.0 mg	2	0.70 (-0.87 to 2.27)	0.383	98.7	< 0.001
	10.0 mg	3	-0.63 (-1.44 to 0.17)	0.122	96.3	< 0.001
Empagliflozin	10.0 mg	4	-1.02 (-2.33 to 0.28)	0.124	99.5	< 0.001
	25.0 mg	4	-1.62 (-2.68 to -0.57)	0.003	99.1	< 0.001
Ipragliflozin	50.0 mg	2	3.61 (-5.77 to 12.99)	0.451	99.3	< 0.001
Overall	-	17	-0.23 (-0.82 to 0.36)	0.446	99.1	< 0.001

Information Classification: General

6. BUN (mg/dL)

Table 1. The summary results for the changes of BUN (mg/dL)

Drugs	Dose	No. of cohorts	WMD and 95%CI	*P* value	I^2 (%)	*P* value for I^2
Canagliflozin	50.0 mg	1	0.75 (0.59 to 0.91)	< 0.001	-	-
	100.0 mg	1	0.87 (0.72 to 1.02)	< 0.001	-	-
	200.0 mg	1	0.68 (0.54 to 0.82)	< 0.001	-	-
	300.0 mg	2	1.14 (-0.04 to 2.33)	0.059	99.0	< 0.001
Dapagliflozin	2.5 mg	4	1.25 (0.88 to 1.62)	< 0.001	98.1	< 0.001
	5.0 mg	6	1.41 (1.06 to 1.75)	< 0.001	98.7	< 0.001
	10.0 mg	6	1.61 (1.20 to 2.02)	< 0.001	99.1	< 0.001
	20.0 mg	1	1.83 (1.68 to 1.98)	< 0.001	-	-
	50.0 mg	1	2.28 (2.12 to 2.44)	< 0.001	-	-
Ipragliflozin	50.0 mg	1	1.68 (1.52 to 1.84)	< 0.001	-	-
Overall	-	24	1.39 (1.20 to 1.59)	< 0.001	98.7	< 0.001

7. Urinary glucose/creatinine ratio (g/g)

Table 1. The summary results for the changes of urinary glucose/creatinine ratio (g/g)

Drugs	Dose	No. of cohorts	WMD and 95%CI	*P* value	I^2 (%)	*P* value for I^2
Dapagliflozin	1.0 mg	1	37.26 (34.72 to 39.80)	< 0.001	-	-
	2.5 mg	4	28.20 (16.16 to 40.24)	< 0.001	99.9	< 0.001
	5.0 mg	6	34.84 (28.01 to 41.67)	< 0.001	99.7	< 0.001
	10.0 mg	6	42.71 (37.28 to 48.13)	< 0.001	99.6	< 0.001
Overall	-	17	36.21 (31.50 to 40.92)	< 0.001	99.8	< 0.001

Information Classification: General
